# Health-Related Lifestyles in Relation to Body Mass Index Among Young and Middle-Aged Women in Japan

**DOI:** 10.1089/whr.2022.0049

**Published:** 2022-11-01

**Authors:** Rupa Singh, Carolyn K. Nyamasege, Steven R. Hawks, Yukiko Wagatsuma

**Affiliations:** ^1^ Department of Clinical Trials and Clinical Epidemiology, Graduate School of Comprehensive Human Sciences, University of Tsukuba, Tsukuba, Japan.; ^2^ Department of Clinical Trials and Clinical Epidemiology, Faculty of Medicine, University of Tsukuba, Tsukuba, Japan.; ^3^ Kinesiology and Health Science, Utah State University, Logan, Utah, USA.

**Keywords:** Japan, middle-aged women, overweight/obesity, underweight

## Abstract

**Background:**

Being underweight, overweight, or obese can lead to adverse health effects. Hence, it is important to understand the specific factors that change the burden of underweight and overweight to target appropriate disease control strategies. This study was designed to examine the prevalence and factors associated with underweight and overweight among young and middle-aged women in Japan.

**Materials and Methods:**

A cross-sectional study was conducted among women aged 20–59 years who participated in health checkups at a regional health care center in 2018 and 2019 (*N* = 1722). The assessments included anthropometric, blood pressure measurements, and a standardized self-administered questionnaire. Multivariable logistic regression analysis assessed lifestyle factors associated with body mass index for underweight <18.5 kg/m^2^) and overweight/obesity (25.0 kg/m^2^ and above).

**Results:**

The prevalence of underweight and overweight/obesity were 12.3%, and 22.5%, respectively. No lifestyle factors were found to be significantly associated with being underweight. Having dinner within 2 hours before bed was positively associated with being overweight/obese [adjusted odds ratio (AOR): 1.448, 95% confidence interval (CI): 1.014–2.068]. Women who gained 10 kg since their 20s were more likely to fall into overweight/obesity category (AOR: 9.674, 95% CI: 1.014–2.068). Women who were using a lipid-lowering medication (AOR: 3.150, CI: 1.892–5.246) were associated with three times higher risk of being overweight/obese. Hypertension and dyslipidemia were significantly associated with overweight/obesity (AOR: 3.094, 95% CI: 2.201–4.351 and AOR: 2.498, 95% CI: 1.831–3.409, respectively).

**Conclusion:**

One in five middle-aged women was overweight or obese, whereas one in eight was underweight. In relation to the prevention of overweight/obesity, specific health promotion messages regarding eating timing should be developed.

## Background

Lifestyle behaviors affect body weight and overall health and are influenced by several social characteristics. Undoubtedly, the incidence of many diseases is related to lifestyle. Unhealthy eating habits, physical inactivity, smoking, and excessive alcohol consumption have been associated with several chronic diseases. Moreover, physical inactivity is identified by the World Health Organization (WHO) as the fourth leading risk factor for mortality globally.^1^ The WHO report stated that physical activity levels decline with increasing age, and women show lower physical activity than men.^2^

Although campaigns promoting healthy lifestyles have been conducted for many years, the number of people with diseases resulting from unhealthy behaviors is constantly increasing.^3^ In general, Asian women have a lower body mass index (BMI) for the same age group and a lower percentage of body fat than Caucasian women.^4^ Underweight has increased rapidly among young Japanese women owing to a stronger desire to be slim than in other age groups.^5–7^ In Japan, ∼70% of women are trying to lower their weight.^8^ As of 2019, 20.7% of women aged 20–29 years were reported to be underweight. There are many reasons for underweight, such as high metabolism, skipping meals, eating on the run, illness, active lifestyles, and eating food with insufficient nutrition.^9^ Underweight women are at a risk of being anemic and infertile. Being underweight can lead to adverse health effects, including death.^10^ Moreover, the rates of anemia among women of reproductive age have steadily increased in Japan since 2010. However, the mechanisms underlying iron deficiency are not yet fully understood.^11^

With the decline in physical demands at work and activities of daily living over the past several decades,^12^ exercise during leisure time has been emphasized as an integral part of a healthy lifestyle by various public health organizations.^3^ However, the number of overweight/obesity cases is still on the rise. Obesity has been associated with various noncommunicable diseases (NCDs), including type 2 diabetes and cardiovascular diseases.^13^ The relationship between BMI and lifestyle-related outcomes is used in public health to determine the cause of diseases.^14^ The standard WHO BMI classifications from previous studies were applied to this study.^15,16^ Furthermore, it is critical to comprehend how lifestyle choices affect both underweight and overweight/obesity, affecting their respective burdens. Hence, this study focuses on women's health with changes in lifestyles at young and middle age and its effect on BMI. This study was designed to examine the prevalence and factors associated with underweight and overweight/obesity among young and middle-aged women in Japan.

## Materials and Methods

### Study area and population

**FIG. 1.**
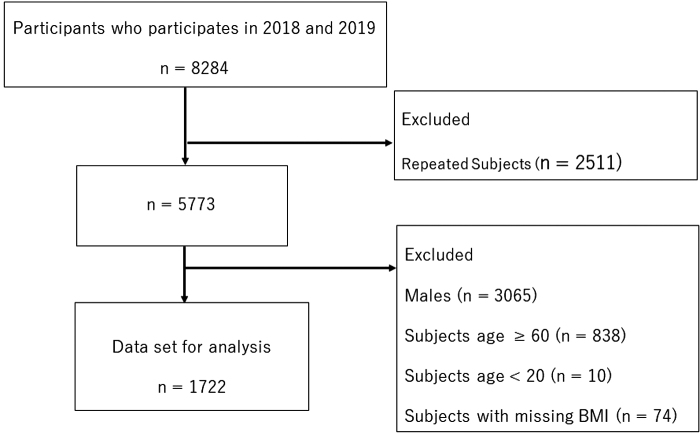
Flowchart of the study population.

The study population included persons older than 20 years of age who participated in medical examination in 2018 and 2019. Some participants repeated the health examination; therefore, only the first examination was used for the analysis. Study participants attended medical examinations at a regional health care center and its outreach sites with an annual attendance of 4270 individuals in 2018 and 4014 individuals in 2019. The health care center conducts medical examinations to promote lifestyle changes and early disease diagnosis, which will help reduce health care costs and increase the quality of life. The study excluded repeated participants, participants who were older than 59 years old, and participants who had missing BMI as given in Figure 1.

### Study design

This study used a cross-sectional design with a focus primarily on women aged between 20 and 59 years.

### Measurements

Anthropometric measurements such as weight (kg), height (cm), waist circumference (cm), and blood pressure (mmHg) were measured by trained personnel at the regional health care center or its outreach sites. Using the WHO's recommended cutoffs, the participants were categorized into three groups based on their BMI: underweight (<18.5 kg/m^2^), normal weight (18.5–24.9 kg/m^2^), and overweight/obesity (≥25.0 kg/m^2^).^17^ Hemoglobin, high-density lipoprotein, low-density lipoprotein cholesterol, and triglycerides were some of the biochemical tests performed at the regional health care center. The cutoff values for hemoglobin (g/dL) <12 and hematocrit (%) <37.0 were used to indicate anemia.

### Questionnaire

A standardized self-administered questionnaire containing 22 items recommended by the Japan Ministry of Health, Labour, and Welfare was used to collect lifestyle-related information and medical history. It included questions on current smoking (smoking in the past month; Yes, No) and weight change since 20s (10 kg), regular physical activity (exercising ≥30 min/session, ≥2 times/week for ≥1 year, or daily walking or physical activity equal to walking ≥1 h/day), enough sleep or not (Yes, No), frequency of alcohol consumption such as sake, shochu, beer, whisky, and so on (rarely, sometimes, or every day), amount of drinking (mL/day), calculated as Japanese sake equivalent (<180, 180–360, 360–540, and >540 mL) and some questions on eating behaviors such as eating speed (fast, normal, or slow), skipping breakfast more than three times a week, having dinner within 2 hours of bedtime ≥3 times/week (Yes, No), taking snacks/sweet drinks in addition to three meals (everyday, sometimes, or rarely). The questionnaire also included information on current health status such as hypertension, dyslipidemia, use of anticholesteric and antidiabetic drug agents and ever diagnosed as having anemia (Yes, No).

### Statistical analysis

Descriptive statistics were used to examine general lifestyle characteristics. The lifestyle behaviors of young women aged ≤39 years were compared with those of middle-aged women aged 40–59 years. BMI, hemoglobin level, hypertension, and dyslipidemia were analyzed as categorical variables. Categorical data are presented as frequencies and percentages, and continuous data are presented as means and standard deviations (SD). The significance of the differences in the parameters was calculated using the Pearson's chi square test. The chi square test was used to identify differences in the proportions of BMIs between the categories of risk factors of the outcome variable (BMI). Logistic regression analysis was conducted to assess the net effect of the exposure variables on the outcome measures. Univariate and multivariable logistic regression analysis were conducted to determine the factors associated with underweight and obesity. Multicollinearity was assessed using variance inflation factors, and the level of significance was set at *p* < 0.05. All analyses were performed using IBM SPSS statistical software version 28.

### Ethical considerations

This study was approved by the Ethics Review Committee of the Faculty of Medicine, University of Tsukuba. All the study procedures were performed in accordance with the Declaration of Helsinki. Informed consent was obtained from all the participants.

## Results

The baseline anthropometric and lifestyle characteristics of the 1722 women are given in Table 1. Obese women (BMI >29.9) were just 5% as given in Table 1; therefore, they were combined with overweight women (25.0 ≤ BMI >29.9) for further analyses. Women below the age of 39 years were classified as young, whereas women between the ages of 40 and 59 years were classified as middle-aged. The mean (SD) age of the women aged 20–39 and 40–59 years was 30.18 ± 5.51 and 50.61 ± 5.65, respectively. A higher proportion (*n* = 1111) of women were within the age group of 40–59 years. In the age group of 20–39 years, 16.2% of women was underweight, whereas it was 10.2% in the age group of 40–50 years. The mean (SD) of hemoglobin (g/dL) was 13.13 ± 1.38 and the mean (SD) hematocrit (%) was 39.75 ± 3.43. The study found that 14.7% of young women had hemoglobin levels <12 g/dL, whereas only 12.7% of middle-aged women had hemoglobin levels <12 g/dL. Similarly, 16.5% of young women had a hematocrit <37.0. Among middle-aged women, approximately one-fourth (26.3%) had hypertension, and one-third (33.1%) had dyslipidemia. Many young and middle-aged women do not use insulin or anti-diabetes, or anti-cholesterol drugs. A proportion of 29.2% of middle-aged and 18.6% of young women were diagnosed with anemia.

**Table 1.** General Characteristics of Participants by Age Category

Variables	All (*N* = 1722)	20–39 Years (*n* = 611)	40–59 Years (*n* = 1111)
Age (years), mean ± SD	43.36 ± 11.39	30.18 ± 5.51	50.61 ± 5.65
BMI (kg/m^2^), mean ± SD	22.51 ± 4.02	22.05 ± 4.27	22.77 ± 3.85
BMI <18.5	212 (12.3)	99 (16.2)	113 (10.2)
18.5 ≤ BMI <25.0	1123 (65.2)	403 (66.0)	720 (64.8)
25.0 ≤ BMI <30.0	299 (17.4)	76 (12.4)	223 (20.1)
BMI ≥30.0	88 (5.1)	33 (5.4)	55 (5.0)
Hemoglobin (g/dL), mean ± SD^a^	13.13 ± 1.38	13.07 ± 1.23	13.15 ± 1.45
<12	224 (13.4)	83 (14.7)	141 (12.7)
≥12	1451(86.6)	483 (85.3)	968 (87.3)
Hematocrit (%), mean ± SD^b^	39.75 ± 3.43	39.57 ± 3.08	39.84 ± 3.59
<37.0	263 (15.7)	93 (16.5)	470 (15.3)
≥37.0	1408 (84.3)	170 (83.5)	938 (84.7)
Hypertension	333 (19.3)	41 (6.7)	292 (26.3)
Dyslipidemia	463 (26.9)	95 (15.5)	368 (33.1)
Diagnosed as having anemia^c^			
Yes	406 (26.1)	85 (18.6)	321 (29.2)
No	1152 (73.9)	373 (81.4)	779 (70.8)
Using antihypertensive drug			
Yes	124 (8.0)	4 (0.9)	120 (10.9)
No	1558 (92.0)	454 (99.1)	980 (89.1)
Using lipid-lowering medication^c^			
Yes	108 (6.9)	5 (1.1)	103 (9.4)
No	1450 (93.1)	453 (98.9)	997 (90.6)

^a^ Missing (*n* = 51).

^b^ Missing (*n* = 47).

^c^ Missing (*n* = 164).

BMI, body mass index; SD, standard deviation.

Table 2 provides the health-related lifestyle variables of women according to BMI categories, such as underweight, normal weight, and overweight/obesity. Only 29.7% of overweight/obese were engaged in regular physical activity compared with those with normal weight (31.4%) and those who were underweight (33.2%). Smoking and daily drinking behaviors among women were 11.7% and 9.9%, respectively. Only 5% of overweight/obese women consume alcohol on daily basis. Approximately 68.5% of the drinkers consumed <180 mL/day. The majority of women reported sufficient sleep (63.5%). Eating behaviors “Eat faster than others” and “Having dinner within 2 hours before bed” were significantly different among underweight, normal, and overweight/obesity.

**Table 2.** Health-Related Lifestyles by Body Mass Index Category

Variables	Underweight	Normal weight	Overweight/obesity	*p*
	BMI <18.5; 212 (12.3%)	BMI: 18.5–24.9; 1123 (65.2%)	BMI ≥25.0; 387 (22.5%)	
Regular physical activity^a^				
Yes	62 (33.2)	317 (31.4)	106 (29.7)	0.695
No	125 (66.8)	693 (68.6)	251 (70.3)	
Smoking^b^				
Yes	22 (11.6)	113 (11.2)	47 (13.2)	0.603
No	168 (88.4)	898 (88.8)	310 (86.8)	
Alcohol consumption^c^				
Everyday	24 (12.6)	110 (10.9)	19 (5.4)	0.007
Sometimes	47 (24.7)	284 (28.1)	89 (25.1)	
None	119 (62.6)	615 (61.0)	246 (69.5)	
Amount of drinking (mL/day)^d^				
<180	82 (68.9)	403 (68.3)	134 (68.7)	0.436
180 − 360	25 (21.0)	155 (26.3)	51 (26.2)	
360–540	11 (9.2)	27 (4.6)	8 (4.1)	
>540	1 (0.8)	5 (0.8)	2 (1.0)	
Sufficient sleep^e^				
Yes	123 (65.4)	632 (63.6)	219 (62.4)	0.783
No	65 (34.6)	362 (36.4)	132 (37.6)	
Skip breakfast^f^				
Yes	34 (18.0)	173 (17.2)	63 (17.7)	0.947
No	155 (82.0)	834 (82.8)	292 (82.3)	
Eat faster than others^g^				
Fast	35 (18.5)	260 (25.7)	120 (33.6)	<0.001
Normal	123 (65.1)	639 (63.2)	217(60.8)	
Slow	31 (16.4)	112 (11.1)	20 (5.6)	
Additional snacks/sweet to three meals^h^				
Everyday	52 (27.8)	131 (26.0)	124 (22.3)	0.490
Sometimes	110 (58.8)	588 (58.4)	223 (63.0)	
Rarely	25 (13.4)	157 (15.6)	52 (14.7)	
Having dinner within 2 hours before bed^c^				
Yes	35 (18.5)	203(20.1)	94 (26.5)	0.025
No	154 (81.5)	806 (79.9)	261 (73.5)	
Gain weight by 10 kg since 20s^g^				
Yes	2 (1.1)	189 (18.7)	249 (69.7)	<0.001
No	187 (98.9)	822 (81.3)	189 (30.3)	

^a^ Missing (*n* = 168).

^b^ Missing (*n* = 164).

^c^ Missing (*n* = 169).

^d^ Missing (*n* = 818).

^e^ Missing (*n* = 189).

^f^ Missing (*n* = 171).

^g^ Missing (*n* = 165).

^h^ Missing (*n* = 174).

Table 3 provides the univariate and multivariate analyses for the selected health variables associated with being underweight. The likelihood of being underweight was lower among women aged 40–59 years [odds ratio (OR): 0.639, 95% confidence interval (CI): 0.475–0.859] than among the women aged 20–39 years. In multivariable analysis, underweight women were not likely to have dyslipidemia [adjusted odds ratio (AOR): 0.537, 95% CI: 0.334–0.864]. No lifestyle variables were significantly associated with being underweight.

**Table 3.** Univariate and Multivariable Logistic Regression Analyses with Factors Associated with Underweight

Variable categories	OR [95% CI]	*p*	AOR [95% CI]	*p*
Age, years				
20–39	Ref.		Ref.	
40–59	0.639 [0.475–0.859]	0.003	0.741 [0.526–1.044]	0.086
Hemoglobin <12 (g/dL)	0.908 [0.581–1.418]	0.672		
Regular physical activity	1.084 [0.778–1.512]	0.633		
Smoking	1.041 [0.641–1.691]	0.872		
Alcohol consumption				
Everyday	1.128 [0.695–1.828]	0.626		
Sometimes	0.855 [0.593– 1.233]	0.402		
None	Ref.			
Sufficient sleep	1.084 [0.781–1.503]	0.629		
Eating speed				
Slow	1.438 [1.924–2.237]	0.107	1.279 [0.812–2.017]	0.289
Fast	0.699 [0.468–1.046]	0.081	0.787 [0.521–1.190]	0.257
Normal	Ref.		Ref.	
Having dinner within 2 hours before bed	0.902 [0.606–1.343]	0.613	0.920 [0.608–1.393]	0.695
Gain weight by 10 kg since 20s	0.047 [0.011–0.189]	0.000	0.053 [0.013– 0.217]	<0.001
Using lipid-lowering medication	0.720 [0.322–1.612]	0.424		
Hypertension	0.682 [0.430–1.083]	0.105	0.738 [0.444–1.225]	0.286
Dyslipidemia	0.486 [0.315–0.749]	<0.001	0.537 [0.334–0.864]	0.013

AOR, adjusted odds ratio; CI, confidence interval; OR, odds ratio; Ref., Reference category.

Table 4 presents univariate and multivariate logistic regression analyses for selected health variables associated with overweight/obesity. Women who eat faster were more likely to be overweight/obese (OR: 1.359, 95% CI: 1.042–1.772). In addition, women having dinner within 2 hours before bed were more likely to be overweight/obese (OR: 1.430, 95% CI: 1.079–1.895). Women who gained 10 kg of body weight since their 20s were 10 times more likely to be overweight/obese (OR: 10.027, 95% CI: 7.611–13.210). In addition, using lipid-lowering medication, hypertension, and dyslipidemia were positively associated with overweight/obesity. While adjusting the listed variables in Table 4, women having dinner within 2 hours before bed were positively associated with overweight/obesity (AOR: 1.448, 95% CI: 1.014–2.068). Women who gained 10 kg since 20s were more likely to be overweight/obese, so finding a positive relationship (AOR: 9.674, 95% CI: 7.135–13.108). Women who were using lipid-lowering medication (AOR: 3.150, 95% CI: 1.892–5.246) were associated with being overweight/obese. Hypertension and dyslipidemia were significantly associated with overweight/obesity (AOR: 3.094, 95% CI: 2.201–4.351 and AOR: 2.498, 95% CI: 1.831–3.409, respectively).

**Table 4.** Univariate and Multivariable Logistic Regression Analyses with Factors Associated with Overweight/Obesity

Variable categories	OR [95% CI]	*p*	AOR [95% CI]	*p*
Age				
20–39	Ref.		Ref.	
40–59	1.428 [1.109–1.838]	0.006	0.649 [0.452–0.933]	0.019
Hemoglobin <12 (g/dL)	0.920 [0.649–1.304]	0.639		
Regular physical activity	0.923 [0.710–1.201]	0.552		
Smoking	1.205 [0.837–1.734]	0.315		
Sufficient sleep	0.950 [0.739–1.222]	0.691		
Alcohol consumption				
Everyday	0.432 [0.260–0.718]	0.001	0.321 [0.174–0.594]	<0.001
Sometimes	0.783 [0.592–1.037]	0.088	0.903 [0.643–1.269]	0.558
None	Ref.		Ref.	
Eating speed				
Fast	1.359 [1.042–1.772]	0.024	1.122 [0.808–1.558]	0.493
Slow	0.526 [0.319–0.867]	0.012	0.695 [0.386–1.253]	0.227
Normal	Ref.		Ref.	
Having dinner 2 hours before bed	1.430 [1.079–1.895]	0.013	1.448 [1.014–2.068]	0.042
Gain weight by 10 kg since 20s	10.027 [7.611–13.210]	<0.001	9.674 [7.135–13.108]	<0.001
Using lipid-lowering medication	3.066 [2.033–4.623]	<0.001	3.150 [1.892–5.246]	<0.001
Hypertension	3.177 [2.441–4.137]	<0.001	3.094 [2.201–4.351]	<0.001
Dyslipidemia	3.215 [2.519–4.103]	<0.001	2.498 [1.831–3.409]	<0.001

AORs have been adjusted for all variables listed.

Ref., Reference category.

## Discussion

This study was designed to assess lifestyle behaviors and BMI in young and middle-aged women between the ages of 20 and 59 years to determine their health-related lifestyle. The overall prevalence of underweight women was 12.3%, whereas the prevalence of overweight/obesity was 22.5%. Similarly, the prevalence of underweight among young women aged ≤39 years was 16.2% and overweight/obesity was 17.8%. Our study found a similar prevalence of underweight, as shown in studies from Japan,^18^ Indonesia,^14^ Kenya,^19^ and Maldives.^20^ This study agrees with the findings of the previous study, which suggested that the prevalence of obesity would appear to be low if the higher cutoff were used. Nevertheless, the prevalence of obesity-related health problems in Japan and the Asia-Oceania region is comparable with that in Western cultures.^21^ This was the first study in Japan to examine health-related lifestyle factors related to BMI in women aged 20–59 years. Most of the previous similar studies reported findings on a wide range of age groups from children, college, and university students to young adults and the elderly people.^22–25^

Some studies have reported that eating slowly is linked to being underweight,^26,27^ but our study failed to find such an association. Because the prevalence of underweight has increased among young Japanese people,^25,28^ it is crucial to address the potential contributions of eating speed to not only the risk of being overweight, but also to that of underweight. According to some studies, eating disorders and the assumption that underweight bodies are more ideal have increased in Southeast Asia.^29^ Likewise, another study from Japan showed that stopping to eat quickly prevents additional weight gain in nonoverweight/obese women.^30^ Hence, slowing down the pace of eating, and chewing more slowly may perhaps be an aspect of sensible eating, and may contribute to healthy eating habits. Our findings also show that having dinner within 2 hours before bed is correlated with overweight/obesity. Thus, late night eating, particularly before bed, has received significant attention.^30^ One study conducted in Japan showed that having dinner within 2 hours of bedtime, more than three times a week, could be a risk factor for the development of lifestyle-related diseases.^31^

The study findings suggest, as previously reported,^32,33^ that cardiometabolic comorbidities, including hypertension and dyslipidemia, were associated with overweight/obesity. According to the WHO, 60% of factors related to individual health and quality of life are correlated with lifestyle.^34^ Thus, changes in eating behavior are common among young and middle-aged Japanese women, but the proportion is lower than the Western adult populations.^35^ Poor lifestyle behaviors can lead to excess BMI, resulting in hypertension, which consequently increases the cardiovascular risk.^36^ Women undergo numerous hormonal changes throughout their different stages of life, which have significant effects on lipid metabolism.^33^ However, more studies are needed to identify factors that lead to increased rates of overweight/obesity, which continuously threatens NCDs. A previous study found that those who took a lipid-lowering medication increased their fat intake and caloric intake, along with their BMI, compared with those who did not take it.^37^

A previous study found that underweight women have a lower risk of diseases such as hypertension, dyslipidemia, and chronic kidney disease than those who are overweight or have normal weight.^38^ Being in a generation of women with a high prevalence of underweight at a young age makes someone more likely to be underweight or overweight/obese at an older age, with several health outcomes.^39^ Thus, a healthy diet, physical activity, and adequate sleep are key to attaining and sustaining a healthy weight.

This study also found that a weight gain of ≥10 kg in adulthood was associated with overweight/obesity. Excessive weight gain in middle-aged individuals is related to higher risk of various forms of metabolic diseases.^36^ Understanding the factors underlying weight change is needed to help control the overweight/obesity pandemic. Our findings also showed that alcohol intake is negatively associated with BMI in women. Studies have shown that alcohol consumption has an inverse association with weight gain. This study agrees with a previous study.^38^ In another study, individuals who consumed alcohol more frequently were less likely to be overweight.^40^ The majority (68.5%) of the women in this study consumed <180 mL a day. However, we could not determine whether their drinking was moderate because we did not have detailed information on their consumption based on the types of drinks. Another study comparing overweight and obesity among Asians and Caucasians^41^ found an association between poor sleep quality and obesity; however, our study did not find such an association. Overweight/obesity has been associated with various factors, including physical activity, smoking, and alcohol intake.^42^ Nevertheless, our study showed no associations between physical activity, smoking, and BMI.

### Limitations of the study

This study was constrained by its cross-sectional design owing to which the study cannot conclude the causality of the current findings. Second, apart from blood pressure and physical measurements, all other information was based on self-reporting, which could have biased the responses. Because this study used the standard WHO BMI guidelines, the Modified Asian Indian criteria may yield slightly different results.^43^ Another limitation is that specific food and energy intakes variables were not collected; therefore, we cannot examine whether late-night eating of specific foods was associated with overweight while assessing nutritional factors such as energy intakes. The results of this study revealed some significant eating behavior variables. Further longitudinal studies are necessary to observe the trends in health-related lifestyles and BMI.

## Conclusion

One in five middle-aged women was overweight or obese, whereas one in eight was underweight. Specific health promotion messages regarding the timing and speed of eating should be developed. This is a baseline study to understand the study population's BMI and its relationship with the health-related lifestyle characteristics of young women. Further longitudinal studies based on this population should be conducted to examine the association and trends of lifestyle factors and body composition among underweight, overweight, and obese women.

## Data Availability

Datasets used in this study are available upon reasonable request from the corresponding author.

## Abbreviations Used

AOR: adjusted odds ratio
BMI: body mass index
CI: confidence interval
NCD: noncommunicable disease
OR: odds ratio
SD: standard deviation
WHO: World Health Organization
